# Radiomics for the identification of extraprostatic extension with prostate MRI: a systematic review and meta-analysis

**DOI:** 10.1007/s00330-023-10427-3

**Published:** 2023-11-13

**Authors:** Andrea Ponsiglione, Michele Gambardella, Arnaldo Stanzione, Roberta Green, Valeria Cantoni, Carmela Nappi, Felice Crocetto, Renato Cuocolo, Alberto Cuocolo, Massimo Imbriaco

**Affiliations:** 1 Department of Advanced Biomedical Sciences, University of Naples Federico II, Via Pansini 5, 80131 Naples, Italy; 2 PO Pellegrini ASL Napoli 1 Centro, Naples, Italy; 3 Department of Neurosciences, Human Reproduction and Odontostomatology, University of Naples Federico II, Naples, Italy; 4 Department of Medicine, Surgery and Dentistry, University of Salerno, Baronissi, Italy

**Keywords:** Magnetic resonance imaging, Prostatic neoplasms, Neoplasm staging

## Abstract

**Objectives:**

Extraprostatic extension (EPE) of prostate cancer (PCa) is predicted using clinical nomograms. Incorporating MRI could represent a leap forward, although poor sensitivity and standardization represent unsolved issues. MRI radiomics has been proposed for EPE prediction. The aim of the study was to systematically review the literature and perform a meta-analysis of MRI-based radiomics approaches for EPE prediction.

**Materials and methods:**

Multiple databases were systematically searched for radiomics studies on EPE detection up to June 2022. Methodological quality was appraised according to Quality Assessment of Diagnostic Accuracy Studies 2 (QUADAS-2) tool and radiomics quality score (RQS). The area under the receiver operating characteristic curves (AUC) was pooled to estimate predictive accuracy. A random-effects model estimated overall effect size. Statistical heterogeneity was assessed with *I*^2^ value. Publication bias was evaluated with a funnel plot. Subgroup analyses were performed to explore heterogeneity.

**Results:**

Thirteen studies were included, showing limitations in study design and methodological quality (median RQS 10/36), with high statistical heterogeneity. Pooled AUC for EPE identification was 0.80. In subgroup analysis, test-set and cross-validation-based studies had pooled AUC of 0.85 and 0.89 respectively. Pooled AUC was 0.72 for deep learning (DL)–based and 0.82 for handcrafted radiomics studies and 0.79 and 0.83 for studies with multiple and single scanner data, respectively. Finally, models with the best predictive performance obtained using radiomics features showed pooled AUC of 0.82, while those including clinical data of 0.76.

**Conclusion:**

MRI radiomics–powered models to identify EPE in PCa showed a promising predictive performance overall. However, methodologically robust, clinically driven research evaluating their diagnostic and therapeutic impact is still needed.

**Clinical relevance statement:**

Radiomics might improve the management of prostate cancer patients increasing the value of MRI in the assessment of extraprostatic extension. However, it is imperative that forthcoming research prioritizes confirmation studies and a stronger clinical orientation to solidify these advancements.

**Key Points:**

*• MRI radiomics deserves attention as a tool to overcome the limitations of MRI in prostate cancer local staging.*

*• Pooled AUC was 0.80 for the 13 included studies, with high heterogeneity (84.7%, p < .001), methodological issues, and poor clinical orientation.*

*• Methodologically robust radiomics research needs to focus on increasing MRI sensitivity and bringing added value to clinical nomograms at patient level.*

**Supplementary Information:**

The online version contains supplementary material available at 10.1007/s00330-023-10427-3.

## Introduction

Prostate MRI has reshaped prostate cancer (PCa) diagnostic pathway, as the “MRI-first” approach is increasingly gaining recognition as standard-of-care, with lesion detection to be performed following acknowledged MRI guidelines (Prostate Imaging-Reporting and Data Systems version 2.1 (PI-RADS v2.1)) [1, 2]. As for local staging, MRI-based grading systems for extraprostatic extension (EPE) detection appear promising, with suspicious imaging features mainly represented by tumor capsular contact length, capsular bulge/irregularity, and frank capsular breach [3–7]. Nevertheless, present guidelines still base pre-treatment local staging (cT) assessment exclusively on digital rectal examination findings, with only a weak recommendation to the use of MRI in this setting [8, 9]. This might be partly due to the lack of standardization affecting the ability of radiologists to detect EPE on MRI, as well as to the reported poor sensitivity [3, 10]. The adoption of different clinical nomograms differently incorporating cT, patient’s demographics, and laboratory and biopsy findings is widespread as a tool for EPE prediction and overall risk stratification, but these do not include imaging features [11]. However, in external validation cohorts, multivariate risk calculators accounting for MRI features have shown significantly higher performance compared to clinical nomograms [12, 13].

Novel MRI biomarkers and advanced quantitative techniques have been recently investigated to further enhance the value of MRI for EPE detection and possibly overcome current limits [14–26]. In particular, radiomics is a novel approach that can translate images into valuable quantitative datasets by the analysis of many mathematical parameters describing different MR image properties [27]. Furthermore, the application of artificial intelligence (AI) and machine learning (ML) may improve the discovery of task-specific features such as anatomic localization, tumor contacting, neurovascular bundles, or direct evidence of abnormalities in periprostatic soft tissue. However, AI in medicine is facing a reproducibility crisis which is hindering the translation of radiomics research into clinical practice, with the scientific community advocating for more robust methodology in the field [28–32]. Additionally, radiomics studies should be specifically designed to address unsolved clinical needs. In the case of EPE prediction, this would mean contextualizing the possible added value of radiomics compared to current standard (i.e., cT and/or clinical nomograms) as well as possibly viable alternatives (i.e., conventional MRI approaches).

In this light, we performed a systematic review and meta-analysis aiming to provide insights into MRI-based radiomics approaches for EPE prediction, by estimating their performance, exploring their heterogeneity, summarizing the main factors impacting the diagnostic accuracy, and focusing on those methodological and study design shortcomings that must be addressed to increase their clinical value.

## Materials and methods

This meta-analysis followed the PRISMA (Preferred Reporting Items for Systematic Reviews and Meta-Analyses) statement (supplementary material for PRISMA Checklist) [33]. The review protocol is registered on PROSPERO (CRD42023392319) [34].

### Literature search and study selection

An English literature search was performed using the PubMed, Embase, Scopus, and Web of Science databases to identify articles published until June 30th, 2022. The study search was restricted to data obtained in humans and conducted using the following key words with their variations: “Magnetic Resonance Imaging,” “MRI,” “Prostate cancer,” “Machine Learning,” “Radiomics,” and “Extraprostatic.” The full search strategy is presented in the supplementary materials. The title and abstract of potentially relevant studies were screened for appropriateness before retrieval of the full article by two reviewers (M.G. and A.S.) and disagreements were discussed with a third reviewer (A.P.) to reach a consensus. The full-published reports of the abstracts selected by the reviewers were retrieved and the same reviewers independently performed a second-step selection based on the inclusion criteria; disagreements were resolved by consensus. Furthermore, in accordance with PRISMA guidelines, the bibliographies of retrieved articles were manually reviewed to identify additional items meeting inclusion criteria.

### Data extraction and eligibility

Data from the included studies was collected in a database. Each study was initially identified considering author, journal, and year of publication. Total patient population, number of positive and negative cases, study type, and MRI field strength were recorded. Furthermore, information regarding the radiomics and ML pipeline, MRI sequences included, lesion segmentation details, image and data preprocessing steps, feature extraction, feature selection, algorithm, prediction model, and validation strategy were collected. Additionally, the following details regarding study design were also registered: (1) whether the radiomics predictive model incorporated non-radiomics characteristics and if so their specifics; (2) whether a comparison with clinical nomograms and/or conventional imaging assessment was performed to investigate the added value of radiomics. Finally, relevant accuracy metrics were extracted for the subsequent pooled analysis. No minimum sample size was chosen for inclusion. A study was included if all the following criteria were met: (1) an analysis focused on EPE prediction was presented; (2) information on area under the receiver operating curve (AUC) and total number of positive and negative cases, respectively defined as patients with or without EPE included in the analysis, were reported; (3) clear definition of the dataset used in the study and input data source. For the purposes of data pooling, in case of studies reporting data for either internal or external test-set, we considered them separately, while if different models were built, the one with the best predictive performance was selected. Reviews, editorials, abstracts, animal studies, conference presentations, and studies not focused on the topic of interest, published in languages other than English, or presenting insufficient data for pooling were excluded.

### Data quality assessment

The methodological assessment of the quality of eligible studies was performed by two reviewers independently (A.S. and A.P.), according to the Quality Assessment of Diagnostic Accuracy Studies 2 (QUADAS-2) tool [35] and radiomics quality score (RQS) [36]. The QUADAS-2 tool offers obvious grades of bias and applicability of primary diagnostic accuracy studies. It comprises four significant domains, namely (1) patient selection; (2) index test; (3) reference standard; and (4) the flow and timing. Each domain contains several signal questions used to help judge the risk of bias (low, high, or unclear). The two reviewers completed the screening process independently. Disagreement in the process of answering questions was discussed until consensus was reached. The RQS represents a system of metrics for the overall evaluation of the methodological validity and thoroughness of radiomics-based studies, and has been adopted in different topics, mainly focused on oncology [37, 38]. It consists of 16 items regarding different steps in the workflow of radiomics. The summed total score ranges between − 8 and 36, while the percentage is calculated on a 0–36 scale.

### Statistical analysis

The predictive accuracy (predicting presence of EPE) was quantified using the AUC for the receiver operating characteristic curve analysis. For each of the included studies, the AUC was extracted with corresponding 95% confidence intervals. The AUC standard error was calculated from the total number of positive and negative EPE patients. The *I*^2^ value was used to assess statistical heterogeneity, providing an estimate of the percentage of variability among included studies. *I*^2^ values of 0–25%, 25–50%, 50–75%, and > 75% represent very low, low, medium, and high heterogeneity, respectively. *I*^2^ was calculated as follows:* I*^2^ = 100% × (*Q* − *df*) / *Q*, where *Q* is Cochran’s heterogeneity statistic and *df* the degrees of freedom. The weight of each study was calculated with the inverse variance method, in which the weight given to each study is chosen to be the inverse of the variance of the effect estimate, minimizing the uncertainty of the pooled effect estimate. Pooling of studies was conducted, and effect size assessed using a random-effects model, which allows to estimate the distribution of true effects between studies accounting for heterogeneity. Publication bias was explored using the effective sample size funnel plot described by Egger et al Two-sided *p* values ≤ 0.05 were considered statistically significant [39]. Subgroup analyses were also performed in relation to the use of dedicated test-set or not, deep learning (DL) or not, single or multiple scanners, and if the best predictive models only included radiomics features or if they combined radiomics features with clinical data.

## Results

### Study selection and data extraction

**Fig. 1:**
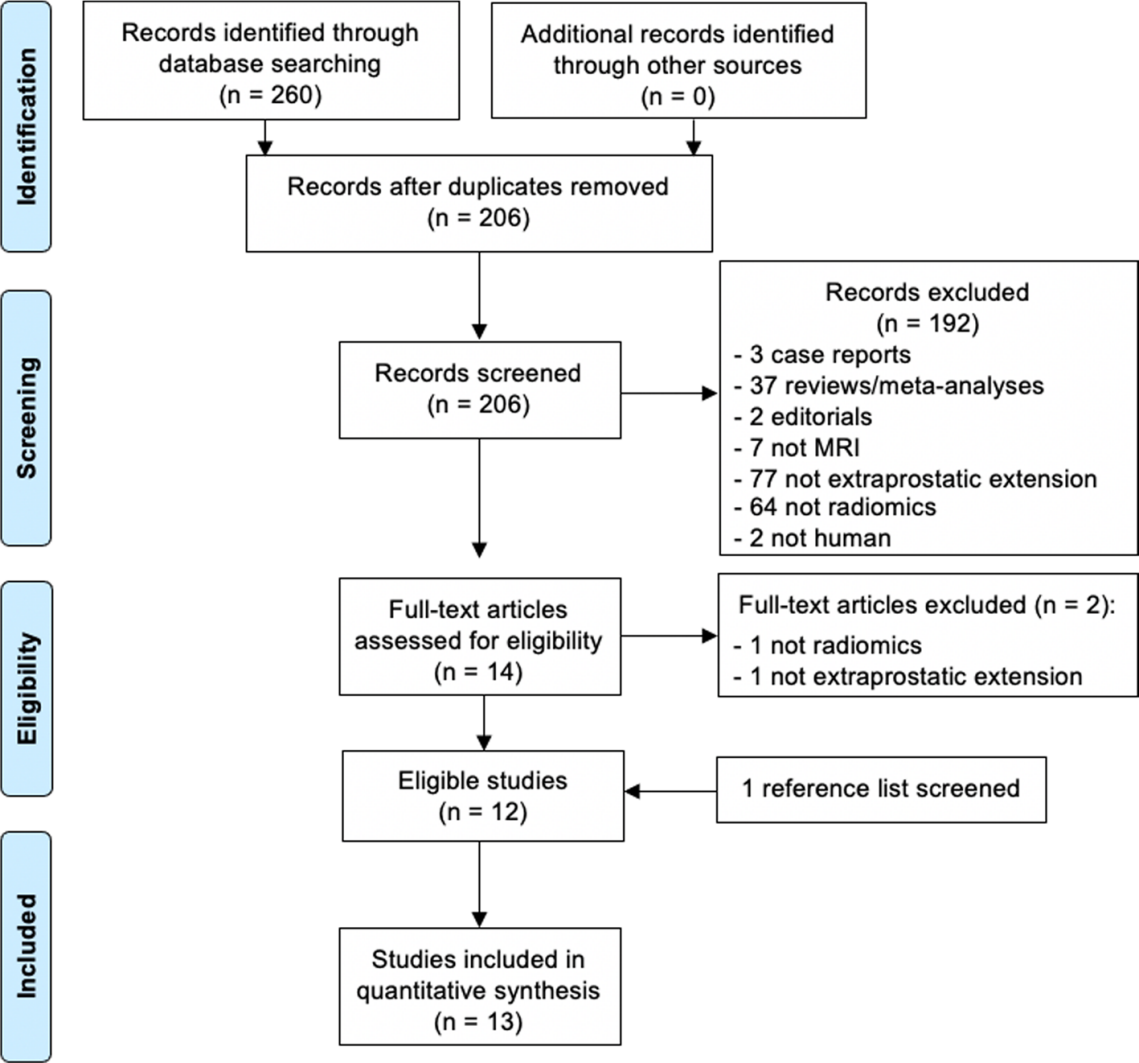
Literature search and study selection process flowchart

The complete literature search process is presented in Fig. 1. In brief, the initial search identified 260 potentially eligible citations. After removing duplicates, 206 records were screened by the reviewers. After the titles and abstracts evaluation, 192 citations were discharged because they were judged to be non-relevant or non-pertinent. Thus, 14 full-text articles were blindly assessed by each investigator for eligibility. After revision, 2 articles were excluded leaving 12 articles. Furthermore, after screening the reference lists of the eligible studies, we identified one additional article that had not been initially captured in our initial search, despite the presence of the selected keywords. Finally, 13 items were the basis of the present meta-analysis [14–26].

The baseline characteristics of the included studies are shown in Tables 1 and 2. Briefly, 3 studies involved more than one institution [14, 15, 19], while 6 investigations adopted multiple scanners [14, 15, 17, 19, 21, 22]. Regarding MRI field strength, a 3-T scanner was mostly adopted (10/13) [14, 17–19, 21–26], while one study was conducted on both 1.5-T and 3-T scanners [15]. Only 1 study used a semi-automatic lesion segmentation approach [16], while the remaining investigations performed either 3D-manual segmentation [14, 15, 17, 18, 21, 22, 24–26] or a combination of manual and automatic approach [20]. Details regarding the study design are presented in Supplementary Tables 1 and 2. Finally, 9 studies had a dedicated test-set [14, 15, 17–19, 21–23, 26].

**Table 1:** Baseline characteristics of the included studies (1)

	*Study type*	*Field strength*	*EPE (n)*	*No EPE (n)*	*Sequences*	*Lesion segmentation*
Bai [14]	Multi-center/multiple scanners	3.0 T	112	172	T2w, ADC	Manual-3D
Cuocolo [15]	Multi-center/multiple scanners	1.5 T/3.0 T	76	117	T2w, ADC	Manual-3D
Damascelli [16]	Single-center/single scanner	1.5 T	38	24	T2w, ADC	Semi-automatic-3D
Fan [17]	Single-center/multiple scanners	3.0 T	50	182	T2w, DWI, DCE	Manual-3D
He [18]	Single-center/single scanner	3.0 T	113	160	T2w, ADC	Manual-3D
Hou [19]	Multi-center/multiple scanners	3.0 T	142	658	T2w, DWI, ADC	Manual-2D
Losnegård [20]	Single-center/single scanner	1.5 T	86	142	T2w, ADC, DCE	Manual-3D, automatic
Ma [21]	Single-center/multiple scanners	3.0 T	101	109	T2w, DWI, DCE	Manual-3D
Ma [22]	Single-center/multiple scanners	3.0 T	100	138	T2w	Manual-3D
Moroianu [23]	Single-center/single scanner	3.0 T	38	85	T2w, ADC	Manual-2D
Shiradkar [24]	Single-center/single scanner	3.0 T	23	22	T2w	Manual-3D
Stanzione [25]	Single-center/single scanner	3.0 T	16	23	T2w, ADC	Manual-3D
Xu et al [26]	Single-center/single scanner	3.0 T	49	66	T2w, DWI, ADC, DCE	Manual-3D

*EPE*, extraprostatic extension; *T2w*, T2-weighted; *DWI*, diffusion-weighted imaging; *ADC*, apparent diffusion coefficient; *DCE*, dynamic contrast enhanced

**Table 2:** Baseline characteristics of the included studies (2)

	*Feature extraction (software)*	*Image preprocessing*	*Data preprocessing*	*Feature selection*	*Algorithm*	*Model§*	*Validation*	*AUC§*
Bai [14]	First- and higher-order (PyRadiomics)	Resampling, intensity normalization	NR	Feature stability, VA, IA + MRMR	LASSO regression	Combined	Test-set	0.72^^^ 0.68^*^
Cuocolo [15]	First- and higher-order, shape (PyRadiomics)	Resampling, intensity normalization, and discretization	Feature scaling and class balancing	Feature stability, VA, and IA + subset evaluator	SVM	Radiomics	Test-set	0.80^*^ 0.73°
Damascelli [16]	First- and higher-order, shape (3D slicer)	Resampling, intensity standardization, normalization, and discretization	NR	Feature stability, VA	Unsupervised HCA, SVM	Radiomics	Cross-validation	0.98
Fan [17]	First- and higher-order, shape (PyRadiomics)	NR	NR	MRMR, RFE based on RF	RF	Combined	Test-set	0.85
He [18]	First- and higher-order, shape (PyRadiomics)	Intensity normalization	Feature scaling	Feature stability testing, VA, and IA + MRMR	LR	Combined	Test-set	0.73
Hou [19]	Deep radiomics	Resampling, intensity normalization	NR	NA	DL network	Radiomics	Test-set	0.81^^^ 0.73^*^
Losnegård [20]	First- and higher-order (Matlab)	Discretization	NR	RF	RF	Combined	Cross-validation	0.80
Ma [21]	First- and higher-order (Matlab)	NR	NR	Feature stability and Kendall correlation analysis	LASSO regression	Radiomics	Test-set	0.88
Ma [22]	First- and higher-order (Matlab)	Intensity normalization	NR	Feature stability and Kendall correlation analysis	LASSO regression	Radiomics	Test-set	0.82
Moroianu [23]	Deep radiomics	Registration, resampling, intensity standardization, and normalization	NA	NA	DL network	Radiomics	Test-set	0.54
Shiradkar [24]	First- and higher-order (Matlab)	Intensity standardization	Feature scaling	Wilcoxon, IA + JMI	SVM	Radiomics	Cross-validation	0.88
Stanzione [25]	First- and higher-order (PyRadiomics)	Intensity normalization	NR	Subset evaluator	Bayesian Network	Radiomics	Cross-validation	0.88
Xu [26]	First- and higher-order (PyRadiomics)	Resampling	NR	Feature stability + MRMR	LASSO regression	Radiomics	Test-set	0.87

^§^In case of multiple models, the one with the best performance was reported, ^ internal test-set, * external test-set 1, ° external test-set 2

*NR*, not reported; *VA*, variance analysis; *IA*, intensity analysis; *MRMR*, maximum relevance minimum redundancy; *LASSO*, least absolute shrinkage and selection operator; *SVM*, support vector machine; *HCA*, hierarchical clustering analysis; *RFE*, recursive feature elimination; *RF*, random forest; *LR*, logistic regression; *DL*, deep learning

### Data quality assessment

**Fig. 2:**
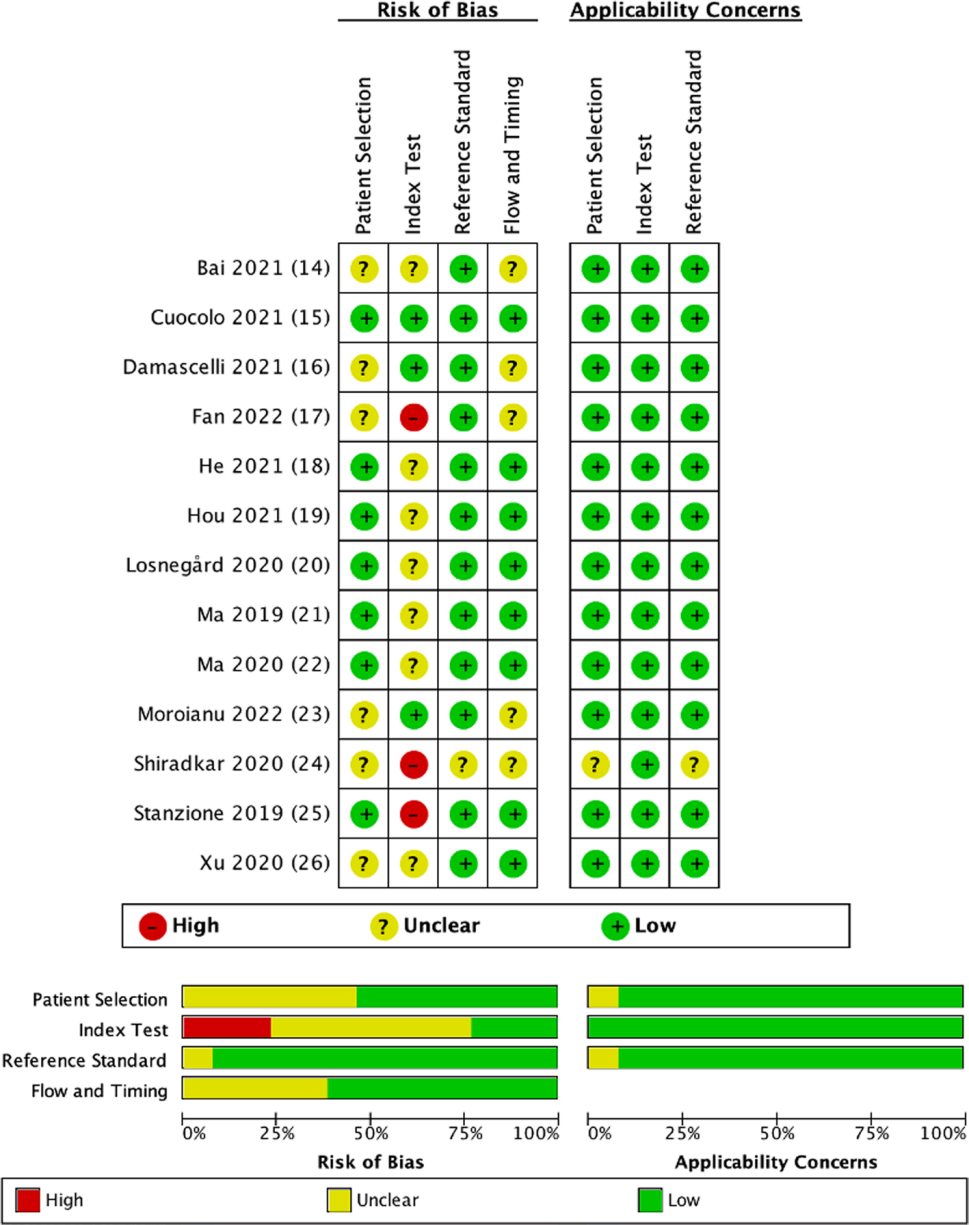
Methodological quality of the included studies assessed according to the Quality Assessment of Diagnostic Accuracy Studies 2 tool for risk of bias and applicability concerns. The green circle represents the low risk of bias, the yellow circle the unclear risk of bias, and the red circle the high risk of bias

The methodological quality assessment of risk of bias within eligible studies according to QUADAS-2 is shown in Fig. 2. In particular, risk of bias due to patient selection was unclear in six studies because a statement on consecutive or random selection was not present [14, 16, 17, 23, 24, 26]. Regarding index test domain, the risk of bias was unclear in seven studies due to the lack of preprocessing details [14, 18–22, 26], while it was high in three studies who did not test feature robustness [17, 24, 25]. Only one study had an unclear risk of bias for providing very few details regarding reference standard [24]. In five cases, the authors did not clearly report the time passed between MRI and radical prostatectomy, receiving an unclear risk of bias [14, 16, 17, 23, 24]. As for applicability concerns, one study scored at unclear risk of bias for the lack of sufficient details regarding patient selection and reference standard [24].

The methodological quality assessment of the included studies according to RQS is shown in Supplementary Table 3. The total RQS ranged from 0 to 33% of the maximum rating, with a median score of 10/36 (interquartile range 11). The RQS was low especially due to the lack of prospective design (all studies were retrospective) and of comparison with gold standard. All investigations performed discriminations statistics while none of them made their code or data publicly available.

### Statistical analysis

**Fig. 3:**
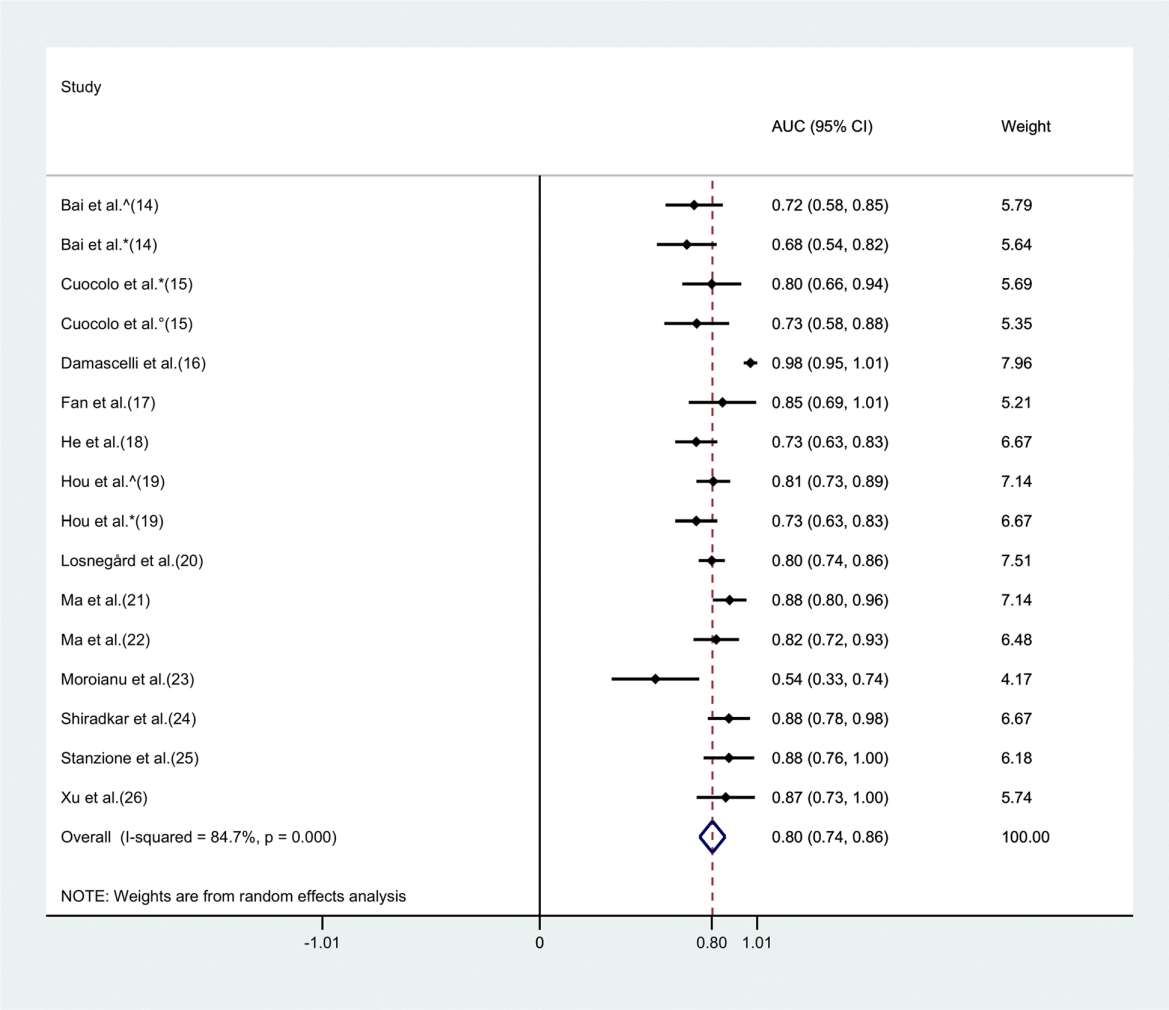
Forest plot of single studies for the pooled area under the curve (AUC) and 95% CI of extraprostatic extension (EPE) characterization. Horizontal lines represent 95% confidence interval of the point estimates. The diamond means the pooled AUC estimate. The red dotted vertical line represents the overall pooled estimate. ^ internal test-set, * external test-set 1, ° external test-set 2

The radiomics models for EPE characterization showed an overall pooled AUC = 0.80 (95% CI = 0.74–0.86) (Fig. 3). Study heterogeneity was 84.7% (*p* < 0.001).

Subgroup analysis was performed to compare studies employing a dedicated test-set within their pipeline and those who did not. Four studies belonged to the latter group with a pooled AUC of 0.89 (95% CI = 0.78–0.99) and heterogeneity of 89.6% (*p* < 0.001). The pooled AUC for the remaining studies was 0.78 (95% CI = 0.73–0.82) and 46.4% heterogeneity (*p* = 0.038). The corresponding plots are presented in Supplementary Fig. 1.

Three studies employed DL within their pipeline for EPE assessment, with a pooled AUC of 0.72 (95% CI = 0.60–0.84) and heterogeneity of 68.4% (*p* = 0.042). The pooled AUC for the studies not adopting DL was 0.82 (95% CI = 0.76–0.89) with heterogeneity of 83.7% (*p* < 0.001). The corresponding plots are presented in Supplementary Fig. 2.

Nine studies employed multiple scanners with a pooled AUC of 0.79 (95% CI = 0.74–0.84) and heterogeneity of 32.6% (*p* = 0.16). The pooled AUC for the investigations adopting single scanners was 0.83 (95% CI = 0.73–0.92) with heterogeneity of 89.8% (*p* < 0.001). The corresponding plots are presented in Supplementary Fig. 3.

Finally, the five studies in which the best predictive performance was achieved by combined models showed a pooled AUC of 0.76 (95% CI = 0.71–0.82) and heterogeneity of 14.8% (*p* = 0.320). The pooled AUC for the remaining studies wherein the best predictive performance was obtained with only-radiomics models was 0.82 (95% CI = 0.75–0.89) with heterogeneity of 84.3% (*p* < 0.001). The corresponding plots are presented in Supplementary Fig. 4.

## Discussion

The present systematic review allowed to identify 13 studies focused on MRI radiomics applications for EPE detection, published between 2019 and 2022, which is in line with the growing overall interest in the field [40, 41]. Most involved more than 100 patients and explored the feasibility of a broad spectrum of different algorithms and models, with none reporting negative results. Nevertheless, limitations in study design and methodological quality were present. In order to be clinically relevant, these studies should seek proofs of an added value of radiomics for EPE prediction over or in addition to MRI and/or currently embraced clinical nomograms. Unfortunately, less than half of the included studies proposed a comparison with conventional MRI assessment as performed by a radiologist while only a single study comparing the radiomics model with a recognized clinical nomogram was found. Similarly, a minority of the studies included clinical data in a holistic radiomics model, and only one presented a fully integrated prediction model obtained combining an established clinical nomogram, the radiologist assessment, and MRI radiomics features [20]. Finally, none of the studies was specifically designed to investigate the possible role of radiomics in filling the previously reported sensitivity gap of conventional MRI assessment for EPE detection [10]. Regarding methodological concerns emerged with QUADAS-2, the shortage of preprocessing details and lack of test feature robustness represent crucial points, as also highlighted by “how to” guides recently published aiming to standardize practice in radiomics [42, 43]. Furthermore, dataset quality should be prioritized to avoid the “garbage-in, garbage-out” phenomenon, for example, by defining the maximum time elapsed between MRI and radical prostatectomy to ensure that EPE cases are reliably classified [32]. Using a dedicated tool (RQS), the overall methodological quality of included studies was found to be heterogeneous, with no prospectively designed studies and sometimes inadequate validation strategies. Neglected items also include investigating biological correlates and publicly sharing data, which might allow researchers to increase the understanding of how radiomics features can play a role for EPE prediction. Indeed, poor explainability is a recognized problem with radiomics, especially compared to more understandable MRI quantitative parameters, like the apparent diffusion coefficient, that have also shown a potential value for EPE prediction [44]. However, low RQS scores should not be intended as a synonym of poor quality but rather as a guide to identify room for improvement; it was also pointed out that DL studies might be penalized by this tool specifically tailored for handcrafted radiomics [45]. In addition to the RQS, there are other checklists currently accessible [46, 47]. For instance, the CLAIM one proves valuable in reporting the modeling components of radiomics research [46]. Furthermore, CLEAR presents a viable alternative, encompassing even more aspects of the studies comprehensively through a single checklist, with a public repository being available to allow the radiomics community to comment on the items and adapt the tool for future versions [47]. All those checklists should be considered to enhance the quality and dependability of radiomics research, and consequently fostering its reproducibility.

Overall, our findings are in line with those of a recent systematic review which included 11 radiomics studies on the topic and similarly underlined the need for greater standardization and rigorousness in methodology [48]. To their merit, the authors of this previous work also included a qualitative synthesis of non-radiomics nomograms for EPE prediction, suggesting a possible added value of MRI to clinical data. Approaching this matter from an alternative perspective, we further expand the knowledge in the field providing a quantitative synthesis of the literature that has shown promising results, with a pooled AUC of 0.80 for EPE prediction MRI-based radiomics models. Different clinical nomograms have been proposed for EPE prediction, including the Memorial Sloan Kettering Cancer Center nomogram and the Partin tables [49, 50]. Nonetheless, these risk stratification tools displayed low accuracy and are strictly correlated with final histopathologic results, with reported AUCs ranging from 0.61 to 0.81 [51]. In a previous meta-analysis, de Rooij et al showed that the pooled sensitivity and specificity were 0.57 and 0.91 for detection of EPE with prostate MRI [10]. However, the included studies assessed EPE with different modalities, including dichotomization, Likert scales, or a standardized lexicon. PI-RADS also addresses EPE, reporting the suspicious features such as tumor contact length, capsule bulging, irregularity, and gross extension, as well as loss of rectoprostatic angle [1]. In a recent meta-analysis of ten studies based on the ESUR PI-RADS scoring system, Li et al reported a pooled AUC of 0.80, with pooled sensitivity and specificity respectively of 0.71 and 0.76 [52]. Furthermore, a 3-point EPE grading system has been recently proposed by Mehralivand and colleagues [53]. In a meta-analysis of four studies using the EPE grading system, Li et al [51] reported a pooled area under the hierarchical summary receiver operating characteristic curve of 0.82. Therefore, our meta-analysis shows that radiomics models align in terms of performance to that of conventional MRI assessment and possibly exceed that of clinical nomograms for the prediction of EPE in PCa, although conflicting data of sensitivity and specificity have been reported.

The subgroup analysis showed lower AUCs for studies with a dedicated (either internal or external) validation set compared to those without that (0.78 vs 0.89). This is unsurprising and widely explained by overfitting, a statistical modeling error that occurs when a function is too closely aligned to a limited set of data [54]. Conversely, a reasonable performance drop is expected when testing a model on an independent external dataset, but this allows to determine its reproducibility and generalizability which are pivotal for clinical translation [55, 56]. Furthermore, the subgroup analysis showed better predictive values for the models using handcrafted radiomics and non-deep ML algorithms compared to those that employed DL (AUC, 0.82 vs 0.72). These results could be at least in part explained by the intrinsic nature of the post-processing ML pipelines, with handcrafted radiomics approaches performing comparably or better than DL algorithm on relatively small-sized datasets [57]. Although new DL algorithms receive much interest from the scientific community, greater attention should be paid to the quality and size of datasets to choose the algorithm that favors the best generalizability of the predictive model. However, it should also be considered that all three studies exploring the predictive performance of DL algorithms had a dedicated test-set. Similar predictive values were found for studies employing single and multiple scanners to assess EPE (AUC of 0.83 and 0.79 respectively). To allow radiomics crossing the translational line between an exploratory investigation method and a standardized added value to precision medicine workflow, more efforts should be done to overcome issues related to multi-scanners and non-uniform scanning parameters from different centers. Finally, the counterintuitive finding of the radiomics-clinical vs radiomics-only models (AUC respectively 0.76 vs 0.82) may be at least partly explained looking at the characteristics of the studies. Indeed, the highest performances of radiomics-only studies were almost exclusively reported in settings with no dedicated test-set [16, 21, 24, 25], with one reaching an AUC as high as 0.98 [16]. Without a proper independent validation, the risk of overly optimistic performance estimates is not negligible. On the other hand, all but one of the radiomics-clinical models [17] were evaluated on a test-set, thus possibly showing lower but more reliable assessments.

Based on the qualitative and quantitative synthesis conducted, we must acknowledge that the primary objective of all the included investigations was to assess new algorithms rather than confirming the predictive performance of previously tested radiomics models. As a result, this has unavoidably led to increased heterogeneity. Given the plethora of available radiomics approaches, we strongly advocate for the necessity of replicative and confirmatory studies to enhance the quality and reliability of radiomics research. Moreover, it is crucial to prioritize the clinical setting wherein radiomics could potentially provide added value. Therefore, radiomics studies should not be limited to technical modeling exercises; instead, they should strive to build compelling evidence and instill confidence in the clinical significance of radiomics.

Our meta-analysis has some limitations to acknowledge. First of all, since accuracy metrics reporting was inconsistent, we were only able to focus on AUC values to perform our meta-analysis. Although previous studies adopted this approach and offer an insight on discriminatory ability [57], pooling additional accuracy metrics would have provided valuable information. Due to the relatively small number of studies included in the quantitative synthesis as well as the high (although expected) heterogeneity, the pooled data should be cautiously interpreted. However, this is a common occurrence in diagnostic accuracy meta-analysis and we explored sources of heterogeneity with the subgroup analyses [58]. Finally, the gray literature was not searched; while some relevant articles might have been missed, gray literature searches are not standardized and source reliability is difficult to prove [59].

In conclusion, radiomics introduces an added layer of complexity to prostate MRI and while it might open an exciting path toward more personalized and precise EPE assessment, its possible role must be brought into context with established tools and more practical alternatives. Technical and diagnostic efficacy studies indicate that radiomics could contribute to redefine how EPE is predicted, alongside radiologist’s evaluation. Methodologically robust research evaluating its diagnostic and therapeutic impact are advocated.

### Supplementary Information

Below is the link to the electronic supplementary material.

Supplementary file1 (PDF 1033 KB)

## Abbreviations

AI: Artificial intelligence
AUC: Area under the curve
CI: Confidence interval
DL: Deep learning
EPE: Extraprostatic extension
ML: Machine learning
PCa: Prostate cancer
QUADAS-2: Quality Assessment of Diagnostic Accuracy Studies 2
RQS: Radiomics quality score

## Declarations

### Guarantor

The scientific guarantor of this publication is Professor Massimo Imbriaco, MD.

### Statistics and biometry

No complex statistical methods were necessary for this paper.

### Methodology

• Systematic review and meta-analysis

• Performed at one institution
