# Supplementary material for: Radiomics for the identification of extraprostatic extension with prostate MRI: a systematic review and meta-analysis
Source: Eur Radiol. 2023 Nov 13;34(6):3981–91. doi: 10.1007/s00330-023-10427-3 (PMC11166859; doi:10.1007/s00330-023-10427-3)
Supplement: Supplementary file 1 — Supplementary file1 (PDF 1033 KB) [file 330_2023_10427_MOESM1_ESM.pdf]

Radiomics for the identification of extraprostatic extension with prostate MRI: a systematic review and meta-analysis

Electronic Supplementary Material

Detailed Search String

(magnetic resonance imaging OR magnetic resonance OR MRI OR MR) AND (Prostate cancer OR prostate tumor) AND (Machine Learning OR radiomics OR texture) AND (Extraprostatic OR extracapsular)

Supplementary Tables

Supplementary Table 1. List of the study proposing combined radiomics/clinical models and details on clinical findings adopted.

Paper	*Clinical data*
Bai [14]	Age, tPSA, biopsy GS
Fan [17]	PSA, WBC, ALP, PLR
He [18]	Age, tPSA, f/tPSA, biopsy GS and positive core percentage
Losnegård [20]	tPSA, TNM stage, biopsy GS and GG, and positive core percentage (MSKCC)
Xu [26]	tPSA and GG

tPSA, total prostate specific agent; GS, Gleason score; WBC, white blood cell; ALP, alkaline phosphatase;

PLR, platelet-to-lymphocyte ration; fPSA, free PSA; GG, Gleason group; MSKCC, Memorial Sloan Kettering Cancer Center

Supplementary Table 2. List of the study presenting formal comparison with alternative approaches, providing details of the comparison.

Paper	*Comparison*	*Diagnostic performance (radiomics/radiomics-clinical)*	*Diagnostic performance (comparison)*	*p value*
Cuocolo [15]	Radiologist assessment	79 %* 74 %°	81 % * 83% °	p = NS
Hou [19]	Radiologist assessment	0.81^ 0.73*	0.73 ^ 0.71 *	$p < .05$
Losnegård [20]	MSKCC and radiologist assessment (EPE grade)	0.80	0.67 § 0.74 “	$p < .05$ $p < .05$
Ma (2019) [21]	Radiologist assessment	0.88	0.6-0.7	$p < .05$
Moroianu [23]	Radiologist assessment	0.54	0.63	NR
Shiradkar [24]	Clinical data (age, PSA, race), biopsy and surgical specimen GGG, prostate volume and PI-RADS v2	0.88	0.53-0.74	NR

Diagnostic accuracies are reported as percentage while AUC values on a 0-1 scale

* external test-set 1; ° external test-set 2; ^ internal test-set; § MSKCC; “ Radiologist assessment; NS, not statistically significant; MSKCC, Memorial Sloan Kettering Cancer Center; PSA, prostate specific agent; GGG, Gleason grade groups; NR, not reported

Supplementary Table 3. Radiomic Quality Scores for all included articles. The total score ranges from −8 to 36, while the percentage is calculated on a 0-36 scale.

**Item**	**Bai [14]**	**Cuocolo [15]**	**Damascelli [16]**	**Fan [17]**	**He [18]**	**Hou [19]**	**Losnegård [20]**	**Ma [21]**	**Ma [22]**	**Moroianu [23]**	**Shiradkar [24]**	**Stanzione [25]**	**Xu [26]**
Image protocol quality	1	1	1	1	1	1	1	1	1	1	1	1	1
Multiple segmentations	1	1	1	0	1	0	0	1	1	0	0	0	1
Phantom study	0	0	0	0	0	0	0	0	0	0	0	0	0
Imaging at multiple time points	0	0	0	0	0	0	0	0	0	0	0	0	0
Feature reduction or adjustment	3	3	3	3	3	-3	3	3	3	-3	3	3	3

Multivariable analysis with non-radiomics features	1	0	0	1	1	1	1	0	0	0	1	0	1
Discuss biological correlate	0	0	0	0	0	0	0	0	0	0	0	0	0
Cut-off analyses	0	0	0	0	1	1	0	0	0	0	0	0	0
Discrimination statistics	1	1	1	1	1	1	1	1	1	1	1	1	1
Calibration statistics	0	1	0	1	0	0	0	2	2	0	0	0	1
Prospective study registered	0	0	0	0	0	0	0	0	0	0	0	0	0
Validation	3	4	-5	2	2	3	-5	2	2	2	-5	-5	2
Comparison to gold standard	0	0	0	0	0	0	2	0	0	0	0	0	0
Potential clinical utility	0	0	0	2	0	2	0	2	2	0	0	0	2

Cost-effectiveness analysis	0	0	0	0	0	0	0	0	0	0	0	0	0
Open science data	0	1	0	0	0	0	0	0	0	0	0	0	0
RQS total	10	12	1	11	10	6	3	12	12	1	1	0	12
RQS (%)	28	33	3	31	28	17	8	33	33	3	3	0	33

RQS = Radiomics Quality Score

Supplementary Figures

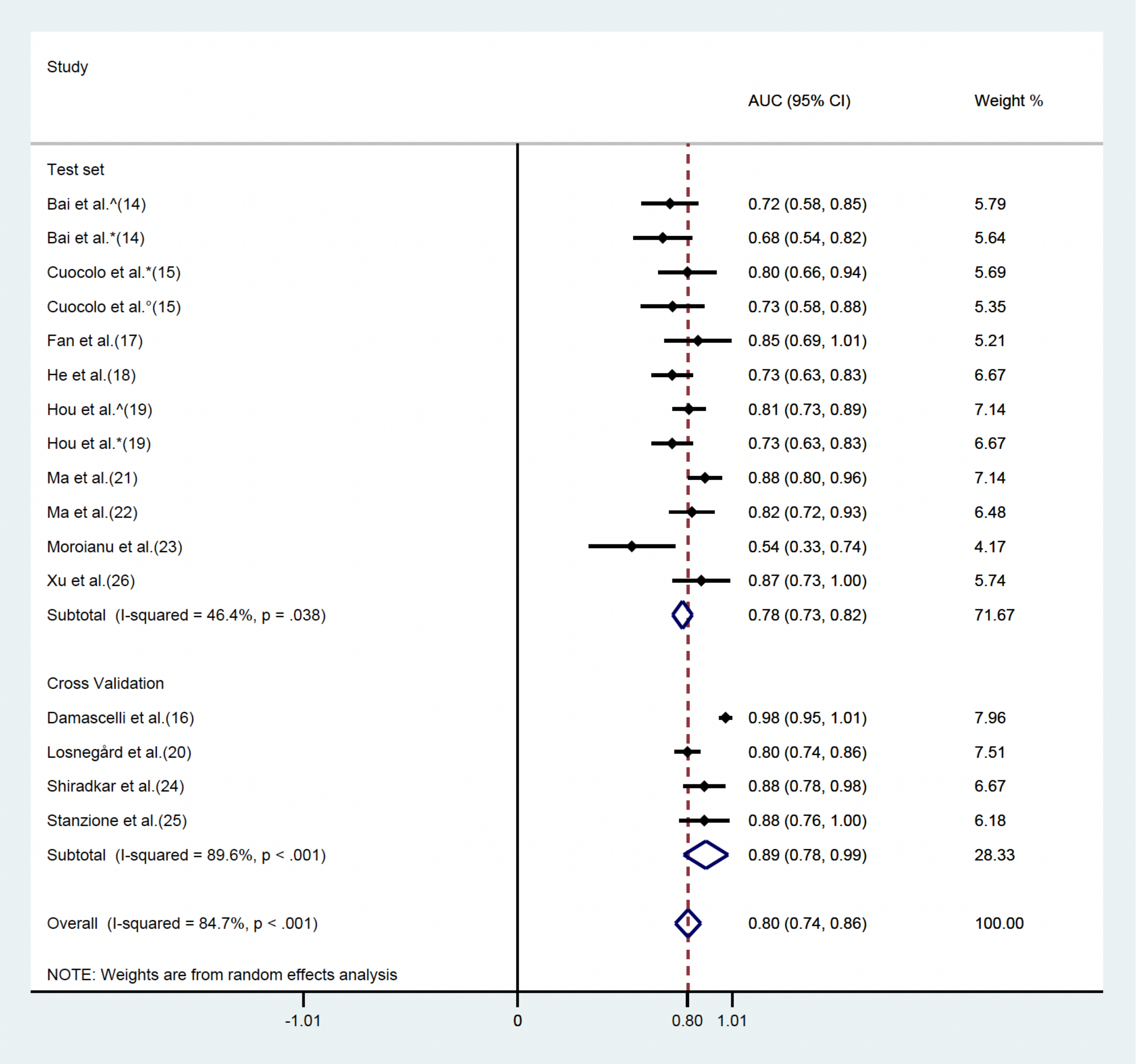

Supplemental Figure 1. Forest plot of single studies for the pooled area under the curve (AUC) and 95% CI of extra-prostatic extension (EPE) characterization employing a dedicated test-set compared to those who did not. Horizontal lines represent 95% confidence interval of the point estimates. The red dotted vertical line represents the overall pooled estimate. The diamond means the pooled AUC estimate. ^ internal test-set, * external test-set 1, ° external test-set 2.

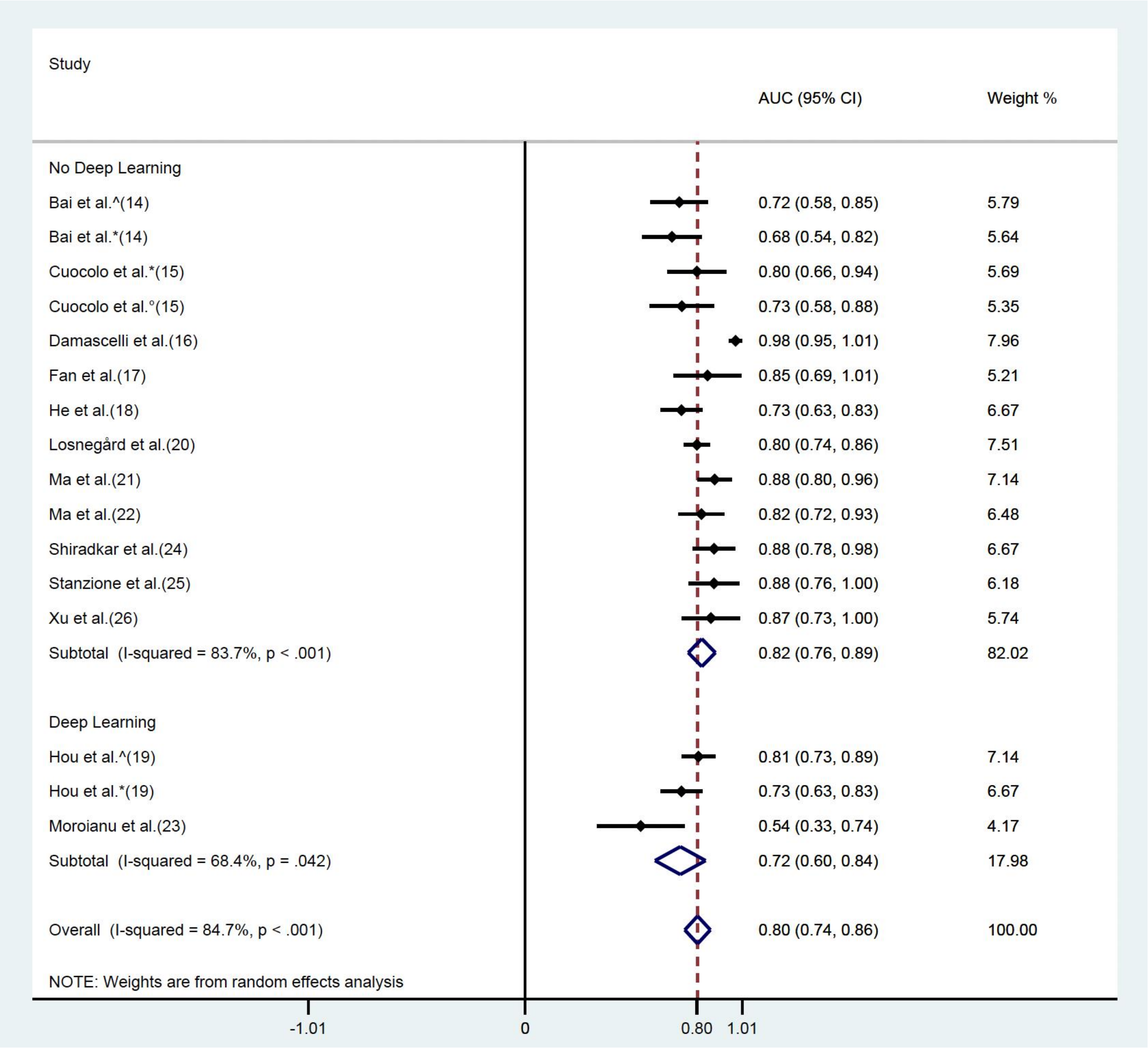

Supplemental Figure 2. Forest plot of single studies for the pooled area under the curve (AUC) and 95% CI of extra-prostatic extension (EPE) characterization using deep learning or not. Horizontal lines represent 95% confidence interval of the point estimates. The diamond means the pooled AUC estimate. The red dotted vertical line represents the overall pooled estimate. ^ internal test-set, * external test-set 1, ° external test-set 2.

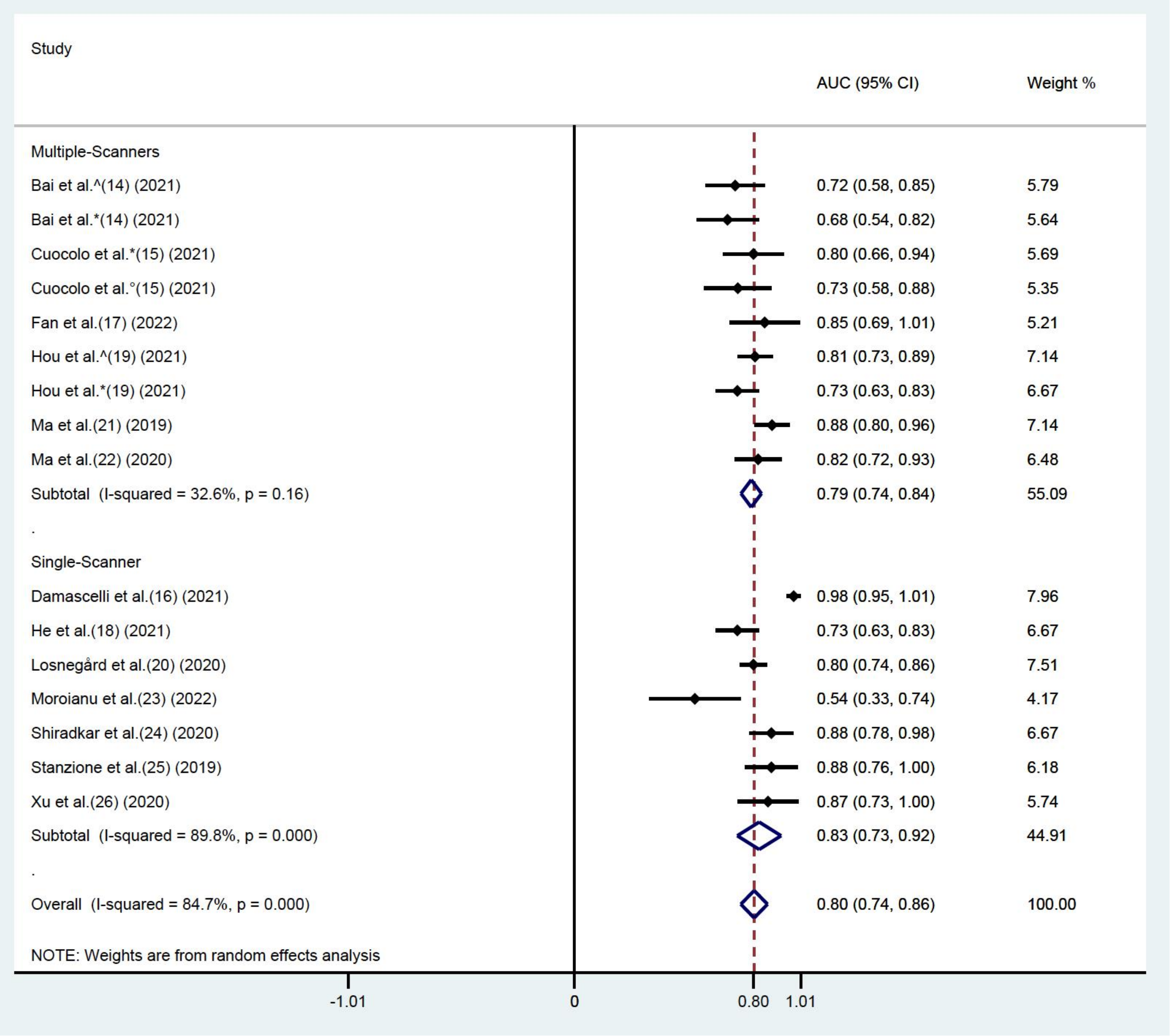

Supplemental Figure 3. Forest plot of single studies for the pooled area under the curve (AUC) and 95% CI of extra-prostatic extension (EPE) characterization employing multiple scanners compared to those employing single scanners. Horizontal lines represent 95% confidence interval of the point estimates. The diamond means the pooled AUC estimate. The red dotted vertical line represents the overall pooled estimate. ^ internal test-set, * external test-set 1, ° external test-set 2.

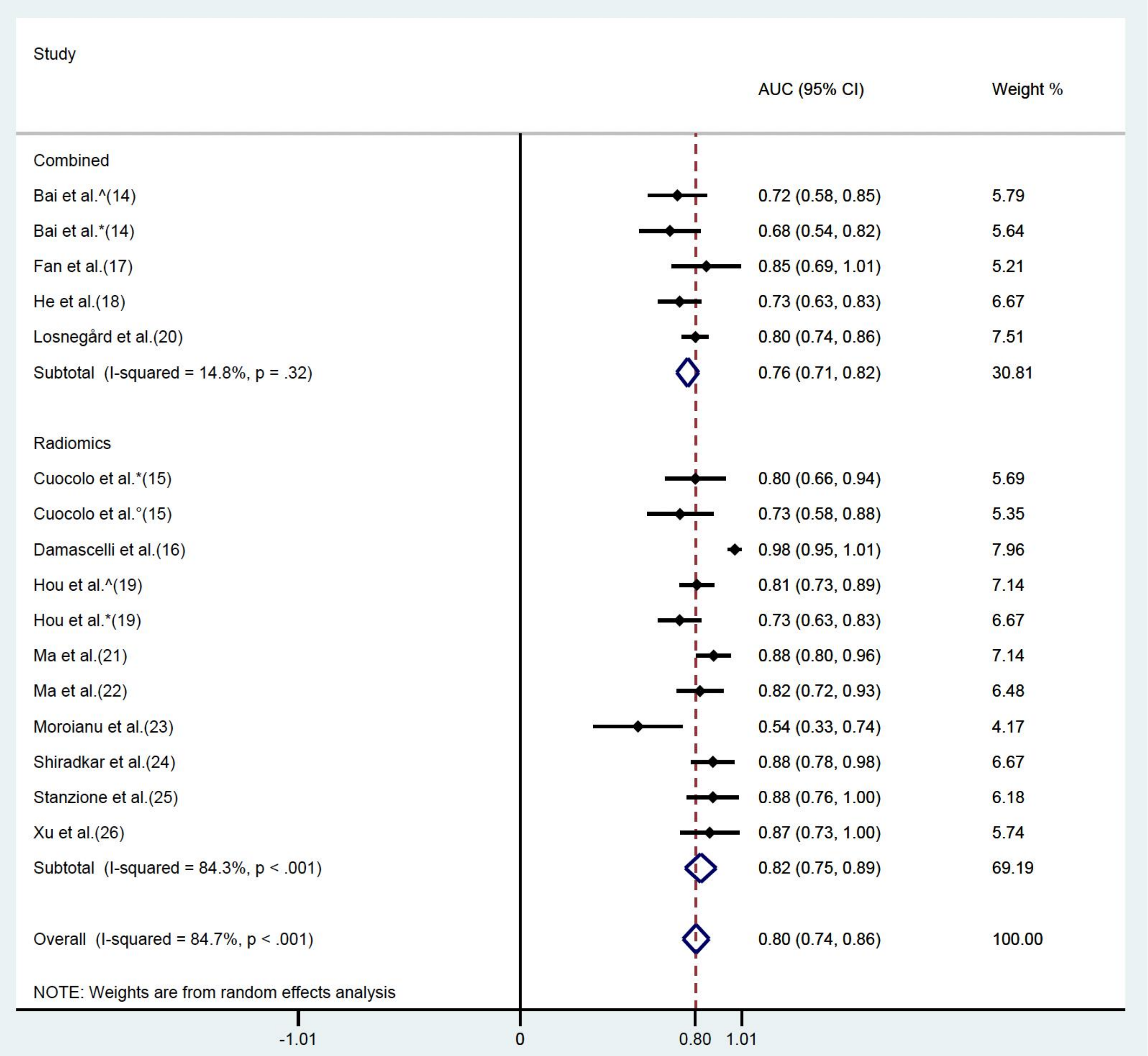

Supplemental Figure 4. Forest plot of single studies for the pooled area under the curve (AUC) and 95% CI of extra-prostatic extension (EPE) characterization in which the best predictive models only included radiomics features compared to those combining radiomics features with clinical data. Horizontal lines represent 95% confidence interval of the point estimates. The diamond means the pooled AUC estimate. The red dotted vertical line represents the overall pooled estimate. ^ internal test-set, * external test-set 1, ° external test-set 2.

PRISMA 2009 Checklist

Section/topic	#	Checklist item	Reported on page #
**TITLE**			
Title	1	Identify the report as a systematic review, meta-analysis, or both.	1
**ABSTRACT**			
Structured summary	2	Provide a structured summary including, as applicable: background; objectives; data sources; study eligibility criteria, participants, and interventions; study appraisal and synthesis methods; results; limitations; conclusions and implications of key findings; systematic review registration number.	1
**INTRODUCTION**			
Rationale	3	Describe the rationale for the review in the context of what is already known.	3-4
Objectives	4	Provide an explicit statement of questions being addressed with reference to participants, interventions, comparisons, outcomes, and study design (PICOS).	-
**METHODS**			
Protocol and registration	5	Indicate if a review protocol exists, if and where it can be accessed (e.g., Web address), and, if available, provide registration information including registration number.	4
Eligibility criteria	6	Specify study characteristics (e.g., PICOS, length of follow-up) and report characteristics (e.g., years considered, language, publication status) used as criteria for eligibility, giving rationale.	4-5
Information sources	7	Describe all information sources (e.g., databases with dates of coverage, contact with study authors to identify additional studies) in the search and date last searched.	4
Search	8	Present full electronic search strategy for at least one database, including any limits used, such that it could be repeated.	Supplementary Material 1
Study selection	9	State the process for selecting studies (i.e., screening, eligibility, included in systematic review, and, if applicable, included in the meta-analysis).	4-6
Data collection process	10	Describe method of data extraction from reports (e.g., piloted forms, independently, in duplicate) and any processes for obtaining and confirming data from investigators.	5
Data items	11	List and define all variables for which data were sought (e.g., PICOS, funding sources) and any assumptions and simplifications made.	5-6
Risk of bias in individual studies	12	Describe methods used for assessing risk of bias of individual studies (including specification of whether this was done at the study or outcome level), and how this information is to be used in any data synthesis.	6
Summary measures	13	State the principal summary measures (e.g., risk ratio, difference in means).	6-7

PRISMA 2009 Checklist

Synthesis of results	14	Describe the methods of handling data and combining results of studies, if done, including measures of consistency (e.g., I^2) for each meta-analysis.	7

Section/topic	#	Checklist item	Reported on page #
Risk of bias across studies	15	Specify any assessment of risk of bias that may affect the cumulative evidence (e.g., publication bias, selective reporting within studies).	7
Additional analyses	16	Describe methods of additional analyses (e.g., sensitivity or subgroup analyses, meta-regression), if done, indicating which were pre-specified.	7
**RESULTS**			
Study selection	17	Give numbers of studies screened, assessed for eligibility, and included in the review, with reasons for exclusions at each stage, ideally with a flow diagram.	7<br>Figure 1
Study characteristics	18	For each study, present characteristics for which data were extracted (e.g., study size, PICOS, follow-up period) and provide the citations.	7-8<br>Table 1-2, supplementary Table 1-2
Risk of bias within studies	19	Present data on risk of bias of each study and, if available, any outcome level assessment (see item 12).	8<br>Figure 2, supplementary Table 3
Results of individual studies	20	For all outcomes considered (benefits or harms), present, for each study: (a) simple summary data for each intervention group (b) effect estimates and confidence intervals, ideally with a forest plot.	Figure 3
Synthesis of results	21	Present results of each meta-analysis done, including confidence intervals and measures of consistency.	9
Risk of bias across studies	22	Present results of any assessment of risk of bias across studies (see Item 15).	9<br>Figure 3
Additional analysis	23	Give results of additional analyses, if done (e.g., sensitivity or subgroup analyses, meta-regression [see Item 16]).	9, supplementary Figures 1-2-3-4
**DISCUSSION**			
Summary of evidence	24	Summarize the main findings including the strength of evidence for each main outcome; consider their relevance to key groups (e.g., healthcare providers, users, and policy makers).	10-12
Limitations	25	Discuss limitations at study and outcome level (e.g., risk of bias), and at review-level (e.g., incomplete retrieval of identified research, reporting bias).	12-13

Conclusions	26	Provide a general interpretation of the results in the context of other evidence, and implications for future research.	13
**FUNDING**			
Funding	27	Describe sources of funding for the systematic review and other support (e.g., supply of data); role of funders for the systematic review.	-

From: Moher D, Liberati A, Tetzlaff J, Altman DG; PRISMA Group. Preferred reporting items for systematic reviews and meta-analyses: the PRISMA statement. PLoS Med. 2009; 6: e1000097.
